# External validation of a clinical risk score to predict hospital admission and in-hospital mortality in COVID-19 patients

**DOI:** 10.1080/07853890.2020.1828616

**Published:** 2020-10-09

**Authors:** Alexandra Halalau, Zaid Imam, Patrick Karabon, Nikhil Mankuzhy, Aciel Shaheen, John Tu, Christopher Carpenter

**Affiliations:** a Internal Medicine Department, Beaumont Health, Royal Oak, MI, USA; b Oakland University William Beaumont School of Medicine, Rochester, MI, USA; c Clinical Informatics, Beaumont Health, Southfield, MI, USA; d Section of Infectious Disease, Beaumont Health, Royal Oak, MI, USA

**Keywords:** Mortality, internal medicine, emergency medicine, COVID-19, corona virus, triage, hospitalization, admission

## Abstract

**Background:**

Identification of patients with novel coronavirus disease 2019 (COVID-19) requiring hospital admission or at high-risk of in-hospital mortality is essential to guide patient triage and to provide timely treatment for higher risk hospitalized patients.

**Methods:**

A retrospective multi-centre (8 hospital) cohort at Beaumont Health, Michigan, USA, reporting on COVID-19 patients diagnosed between 1 March and 1 April 2020 was used for score validation. The COVID-19 Risk of Complications Score was automatically computed by the EHR. Multivariate logistic regression models were built to predict hospital admission and in-hospital mortality using individual variables constituting the score. Validation was performed using both discrimination and calibration.

**Results:**

Compared to Green scores, Yellow Scores (OR: 5.72) and Red Scores (OR: 19.1) had significantly higher odds of admission (both *p* < .0001). Similarly, Yellow Scores (OR: 4.73) and Red Scores (OR: 13.3) had significantly higher odds of in-hospital mortality than Green Scores (both *p* < .0001). The cross-validated C-Statistics for the external validation cohort showed good discrimination for both hospital admission (*C* = 0.79 (95% CI: 0.77–0.81)) and in-hospital mortality (*C* = 0.75 (95% CI: 0.71–0.78)).

**Conclusions:**

The COVID-19 Risk of Complications Score predicts the need for hospital admission and in-hospital mortality patients with COVID-19.

**Key points:**
- Can an electronic health record generated risk score predict the risk of hospital admission and in-hospital mortality in patients diagnosed with coronavirus disease 2019 (COVID-19)?
- In both validation cohorts of 2,025 and 1,290 COVID-19, the cross-validated C-Statistics showed good discrimination for both hospital admission (C = 0.79 (95% CI: 0.77–0.81)) and in-hospital mortality (C = 0.75 (95% CI: 0.71–0.78)), respectively.
- The COVID-19 Risk of Complications Score may help predict the need for hospital admission if a patient contracts SARS-CoV-2 infection and in-hospital mortality for a hospitalized patient with COVID-19.

## Introduction

### Background/rationale

Severe acute respiratory syndrome coronavirus-2 (SARS-CoV-2) is a positive-sense RNA virus belonging to the *Coronaviridae* family, first reported in a cluster of patients with viral pneumonia in Wuhan, China [1,2]. Rapid spread ensued and novel coronavirus infections (COVID-19) were declared as a pandemic on 11 March 2020, resulting in global aggressive social distancing measures to limit viral transmission [3]. As of 19 May 2020, there are 4,897,492 confirmed cases of COVID-19 with 323,285 deaths in 188 countries [4].

SARS-CoV-2 primarily spreads *via* respiratory droplets and direct contact [5–7]. Medical procedures that induce aerosol production, such as nebulizer treatments or intubation, are reported to increase the risk of transmission [1,6,7]. A wide clinical spectrum of severity is reported, and worse clinical outcomes are observed with older patients and patients with comorbidities such as hypertension, diabetes, and chronic obstructive lung disease (COPD) [8,9]. Severe cases can result in shock, acute respiratory distress syndrome (ARDS), acute kidney, cardiac, liver, gastrointestinal, neurological injury, coagulopathy and death [10,11].

Despite COVID-19 infection severity in higher risk populations, most drugs have proven no significant efficacy in large-scale studies [12–14], except remdesivir, currently considered the most promising antiviral agent [12,15]. Hospitalized patients with advanced COVID-19 and lung involvement who received remdesivir had a 31% faster recovery than similar patients who received placebo in the Adaptive COVID-19 Trial sponsored by the National Institute of Allergy and Infectious Disease [15]. However, given the lack of widely available effective therapies, COVID-19 continues to be a global health threat with a massive burden on health care systems. Beyond social distancing and personal protective equipment use, intensive care unit (ICU) capacity expansion and treatments to reduce ICU demand are potential strategies to mitigate the pandemic’s impact [16].

Developing and validating clinically applicable prognostic tools to identify high-risk patients is necessary to guide resource allocation efforts. Recently proposed prediction models for COVID-19, derived primarily from populations in China, Italy, and international registries, suffer from high risk of bias due to small sample sizes, model overfitting, and lack of external validation, and are not yet recommended for clinical practice [17,18].

### Objectives

We aimed at validating a risk assessment tool for patients with COVID-19, stratifying patients based on their hospitalization and in-hospital mortality risk.

## Methods

Beaumont Health is the largest health system in Southeast Michigan, USA providing healthcare services to about one third of patients in the Detroit Metropolitan Area [19]. A retrospective cohort was created from patients with positive SARS-CoV-2 testing on nasopharyngeal swabs per WHO definitions [20] between 1 March 2020 and 1 April 2020 presenting to any of Beaumont Health’s eight emergency departments (EDs). COVID-19 confirmed patients who remained hospitalised beyond 12 May 2020 were excluded given the absence of final outcome data in this group. Additionally, ambulatory (clinic) setting testing was not available during the study timeframe, and hence, was not evaluated. Data on the cohort were abstracted using automated reports generated through ToadDataPoint multi-platform database query tool from Beaumont’s electronic health record (EHR) (EPIC System, Verona, WI, USA). The risk score was automatically calculated and reported in Epic, the most commonly utilised EHR platform in the United States [21,22] (see Supplementary Appendix). This study was approved as an exempt retrospective chart review by the Beaumont Health Institutional Review Board.

### Participants

We defined admitted patients as patients with confirmed SARS-CoV-2 infection who required hospital admission to any of the eight Beaumont hospitals. We defined outpatients as patients who were sent home from their initial ED encounter during which a COVID-19 diagnosis was established. To validate the utility of the risk score in triaging patients on their initial visits to the healthcare system, we excluded outpatients who presented to the ED on subsequent encounters and were admitted to the hospital.

### Outcomes

Two outcome variables were measured, both using a yes/no binary scale: hospital admission and in-hospital mortality. Hospital admission on the first encounter to the ED was evaluated for the entire cohort, while mortality was evaluated only for inpatients who were discharged prior to 12 May 2020. Mortality was evaluated only for the duration of the COVID-19 hospitalization and out of hospital mortality was not evaluated.

### Risk of COVID-19 complications score (risk assessment tool)

The risk score components are: (i) age divided into four categories, <60 years old, 60–69 years old, 70–79 years old and ≥ 80 years old; (ii) male sex; (iii) the presence of coronary artery disease; (iv) the presence of congenital heart disease (v) the presence of congestive heart failure; (vi) the presence of end-stage renal disease (ESRD); (vii) the presence of end-stage liver disease (ESLD); (viii) the presence of chronic pulmonary disease (such as pulmonary fibrosis/chronic obstructive pulmonary disease/bronchial asthma); (ix) the presence of diabetes; (x) the presence of hypertension; (xi) the presence of obesity; (xii) nursing home residence; (xii) pregnancy status; and (xiii) immunocompromised status defined by one of: (a) diagnosis of human immunodeficiency virus (HIV) infection, (b) actively receiving chemotherapy, (c) receiving immunosuppressive agents, (d) carrying a diagnosis of iatrogenic immunosuppression. The items included in the score are automatically retrieved by the EHR from different areas of the patient’s chart, including problem lists and local hospital registries for chronic conditions, computed, and entered into the patient’s record. The maximum score is 15 and each of the 12 items reported receives 1 point if present, apart from age where a patient <60 years old receives 0 points, 60–69 years old receives 1 point, 70–79 years old receives 2 points, and >80 years old receives 3 points (Supplementary Appendix). The score is subsequently divided into three risk categories: (i) green (score 0–2), (ii) yellow (score 3–5), (iii) red (score 6–15), and once validated, it is meant to be available for providers to view and aid in triage decisions in both outpatient and inpatient settings.

The risk score was developed by Dr. David Daniel at Confluence Health. The elements of the risk score were derived from the guidance published by the Centre for Disease Control (CDC) in the United States on conditions that increase the risk of severe illness from COVID-19 for all patients [23]. The risk score was not created from a validation cohort, was only based on expert opinion of the data available. Given the lack of a prior validation cohort from which these variables were given weights, we sought to evaluate the initially selected components of the risk score. No modifications in weight assignment (defined by number of points assigned to each risk factor) were made given the absence of a secondary cohort to validate these modifications.

### Bias

We included all available patients in the final sample size to minimise selection bias, only excluding patients who did not have an outcome at the time of the analysis and those who were initially discharged from the ED and then returned for re-evaluation. Ascertainment bias was limited *via* automated reports data collection.

### Study size

We did not calculate a sample size as we included in our cohort all the patients that met the inclusion criteria as of 1 April 2020.

### Statistical methods

Descriptive statistics were generated for all variables, which were stratified by both admission and mortality with T-Test to compare COVID-19 Risk of Complications Score and Chi-Square tests to compare all other variables. All numbers were rounded to two decimal places. Multivariate logistic regression models were fit for admission on all inpatient and outpatient encounters and in-hospital mortality for inpatient encounters only. All variables incorporated in the Epic COVID-19 Risk of Complications Score were included in the regression for admission. Due to issues with complete separation of some covariates in the external validation dataset, all variables except pregnancy, congenital heart disease, and ESLD were included in the regression for in-hospital mortality. Multivariate logistic regression results are presented in terms of Adjusted Odds Ratios (AOR) with corresponding 95% confidence intervals and *p*-values.

An analysis using an external validation cohort was performed. Discrimination was evaluated using a Cross-Validated C-Statistic, along with its corresponding 95% Confidence Intervals and Receiver Operating Characteristic (ROC) curve. C-Statistics ≥0.7 were considered good and ≥0.8 were considered strong values [24]. Calibration was assessed using the decile method, a method where patients were divided into ten deciles based on their predicted probability for the outcome as predicted from the regressions. Decile calibration plots with superimposed Local Regression-based (LOESS) calibration curves [25] were generated.

In addition to the external validation analysis, the discrimination of the Green/Yellow/Red categorizations also was evaluated using C-Statistics. The C-Statistics for the Green/Yellow/Red categorizations were compared for differences from the full external validation models with a Wald Test.

Any *p*-values <.05 were considered as statistically significant associations. All analysis was done in SAS 9.4 (SAS Institute Inc., Cary, NC, USA).

## Results

There were 2126 encounters with data extracted from Epic (1305 inpatient encounters and 821 outpatient encounters). We excluded 86 outpatient encounters who subsequently returned to ED at a later date, 14 inpatient encounters where the patient was still admitted as of 12 May 2020 and their ultimate disposition was still unknown, and one inpatient encounter where Epic was unable to procure the necessary information to calculate the COVID-19 Risk of Complications Score. The final sample includes 2025 encounters, divided in 1290 hospital admission encounters and 735 outpatient encounters who were never admitted to one of the eight hospitals. Each of these encounters represents a unique patient. Descriptive statistics are shown in Table 1.

**Table 1.** Descriptive statistics of the two external validation cohorts: inpatient plus outpatient and inpatient only.

Variables collected on inpatient and outpatient data	
COVID-19 Risk of Complications Score (*n* = 2025)	
Mean (Standard De*via*tion)	3.19 (2.13)
Median (Interquartile Range)	3.00 (1.00, 4.00)
Minimum Value, Maximum Value	0.00, 11.00
Admission as Inpatient (*n* = 2025)	
Yes	1,290 (63.70%)
No	735 (36.30%)
COVID-19 Risk of Complications Score Categories (*n* = 2025)	
Green (Score 0–2)	858 (42.37%)
Yellow (Score 3–5)	853 (42.12%)
Red (Score 6–15)	314 (15.51%)
Age (*n* = 2025)	
<60 years old	1,116 (55.11%)
60–69 years old	424 (20.94%)
70–79 years old	298 (14.72%)
80 or older	187 (9.23%)
Legal Sex (*n* = 2025)	
Female	998 (49.28%)
Male	1,027 (50.72%)
Medical Conditions (*n* = 2,025)	
Immunocompromised	59 (2.91%)
Congestive Heart Failure (CHF)	140 (6.91%)
Congenital Heart Disease (CHD)	3 (0.15%)
Coronary Artery Disease (CAD)	216 (10.67%)
End-Stage Renal Disease (ESRD)	61 (3.01%)
End-Stage Liver Disease (ESLD)	5 (0.25%)
Chronic Pulmonary Disease	519 (25.63%)
Diabetes	560 (27.65%)
Hypertension	1,022 (50.47%)
Obese	1,151 (56.84%)
Nursing Home Residence	109 (5.38%)
Pregnancy Status	13 (0.64%)
Variables Collected on Inpatient Data Only	
Length of Stay (LOS) (*n* = 1274)	
Mean (Standard De*via*tion)	8.25 (7.29)
Median (Interquartile Range)	6.24 (2.93, 11.01)
Minimum Value, Maximum Value	0.02, 40.40
In-Hospital Mortality (*n* = 1290)	
Yes	223 (17.29%)
No	1,067 (82.71%)

Of all encounters in the external validation dataset, 63.70% (95% CI: 61.60%, 65.80%) were hospital admissions. Of the hospital admissions, 17.29% (95% CI: 15.23%, 19.35%) experienced in-hospital mortality. The majority of patients were <60 years old (55.11%) and there was a nearly even split between males (50.72%) and females (49.28%). Half of all patients had hypertension (50.47%) and were obese (56.84%). Around one quarter of the patients had diabetes (27.65%) and chronic pulmonary disease (25.63%), while one-tenth had CAD (10.67%).

The average length of stay for the hospital admission encounters was 8.25 days. Of those whose discharge information is known, the majority were discharged home (58.07%).

### Outcome data

In the multivariate model to predict admission, older age, male gender, congestive heart failure, end-stage renal disease, chronic pulmonary disease, diabetes mellitus, hypertension, obesity, and nursing home residence were independently associated with admission (all AOR > 1 and *p* < .05). While immunocompromised, congenital heart disease, coronary artery disease, end-stage liver disease, and pregnancy had lower odds of admission, there were no significant differences found (all AOR < 1 and *p* ≥ .05) (Table 2).

**Table 2.** External validation cohort variables, stratified by admission and in-hospital mortality.

	Stratified by Admission			Stratified by In-Hospital Mortality		
	Admitted (*n* = 1290)	Not Admitted (*n* = 735)	*p*-Value	Died (*n* = 223)	Alive (*n* = 1067)	*p*-Value
COVID-19 Risk of Complications Score						
Mean (Standard De*via*tion)	3.95 (2.03)	1.86 (1.58)	<.0001	5.27 (1.94)	3.68 (1.94)	<.0001
Risk of Complications Score Categories						
Green (Score 0–2)	332 (25.74%)	526 (71.56%)	<.0001	13 (5.83%)	319 (29.90%)	<.0001
Yellow (Score 3-5)	668 (51.78%)	185 (25.17%)		108 (48.43%)	560 (52.48%)	
Red (Score 6-15)	290 (22.48%)	24 (3.27%)		102 (45.74%)	188 (17.62%)	
Age						
<60 years old	580 (44.96%)	536 (72.93%)	<.0001	45 (20.18%)	535 (50.14%)	<.0001
60–69 years old	302 (23.41%)	122 (16.60%)		57 (25.56%)	245 (22.96%)	
70–79 years old	238 (18.45%)	60 (8.16%)		68 (30.49%)	170 (15.93%)	
80 or older	170 (13.18%)	17 (2.31%)		53 (23.77%)	117 (10.97%)	
Legal Sex						
Female	597 (46.28%)	401 (54.56%)	.0003	105 (47.09%)	492 (46.11%)	.7907
Male	693 (53.72%)	334 (45.44%)		118 (52.91%)	575 (53.89%)	
Medical Conditions						
Immunocompromised	43 (3.33%)	16 (2.18%)	.1368	14 (6.28%)	29 (2.72%)	.0071
Congestive Heart Failure (CHF)	130 (10.08%)	10 (1.36%)	<.0001	37 (16.59%)	93 (8.72%)	.0004
Congenital Heart Disease (CHD)	1 (0.08%)	2 (0.27%)	.2995	0 (0.00%)	1 (0.09%)	.8271
Coronary Artery Disease (CAD)	187 (14.50%)	29 (3.95%)	<.0001	55 (24.66%)	132 (12.37%)	<.0001
End-Stage Renal Disease (ESRD)	56 (4.34%)	5 (0.69%)	<.0001	17 (7.62%)	39 (3.66%)	.0082
End-Stage Liver Disease (ESLD)	4 (0.31%)	1 (0.14%)	.6590	4 (1.79%)	0 (0.00%)	<.0001
Chronic Pulmonary Disease	435 (33.72%)	84 (11.43%)	<.0001	132 (59.19%)	303 (28.40%)	<.0001
Diabetes	478 (37.05%)	82 (11.16%)	<.0001	105 (47.09%)	373 (34.96%)	.0006
Hypertension	832 (64.50%)	190 (25.85%)	<.0001	160 (71.75%)	672 (62.98%)	.0128
Obese	848 (65.74%)	303 (41.22%)	<.0001	145 (65.02%)	703 (65.89%)	.8049
Nursing Home Residence	102 (7.91%)	7 (0.95%)	<.0001	37 (16.59%)	65 (6.09%)	<.0001
Pregnancy Status	3 (0.23%)	10 (1.36%)	.0022	0 (0.00%)	3 (0.28%)	.4279

For prediction of in-hospital mortality in the multivariate model, older age, end-stage renal disease, chronic pulmonary disease, and nursing home residence were significantly associated with in-hospital mortality (all AOR > 1 and *p* < .05). Other variables that had greater odds, but were not significantly associated with in-hospital mortality, included male gender, immunocompromised status, congestive heart failure, coronary artery disease, diabetes, and obesity (all AOR > 1 and *p* ≥ .05). Hypertension had lower odds of in-hospital mortality, but did not meet statistical significance (AOR = 0.70; *p* = .0607) (Table 2).

When reducing the risk score algorithm to categories, the categories were highly predictive of both admission and in-hospital mortality. Compared to Green scores, Yellow Scores (OR: 5.72) and Red Scores (OR: 19.1) had significantly higher odds of admission (both *p* < .0001). Similarly, Yellow Scores (OR: 4.73) and Red Scores (OR: 13.3) had significantly higher odds of in-hospital mortality than Green Scores (both *p* < .0001) (Tables 3 and 4).

**Table 3.** Model predicting admission in green/yellow/red categories.

	OR (95% CI)	*p*-Value
COVID-19 Risk of Complications Score Categories		
Red (Score 6–15)	19.1 (12.3, 29.7)	<.0001
Yellow (Score 3–5)	5.72 (4.62, 7.08)	<.0001
Green (Score 0–2)	Reference Group	

**Table 4.** Model predicting in-hospital mortality in green/yellow/red categories.

	OR (95% CI)	*p*-Value
COVID-19 Risk of Complications Score Categories		
Red (Score 6–15)	13.3 (7.28, 24.4)	<.0001
Yellow (Score 3–5)	4.73 (2.62, 8.55)	<.0001
Green (Score 0–2)	Reference Group	

### Main results

**Figure 1.**
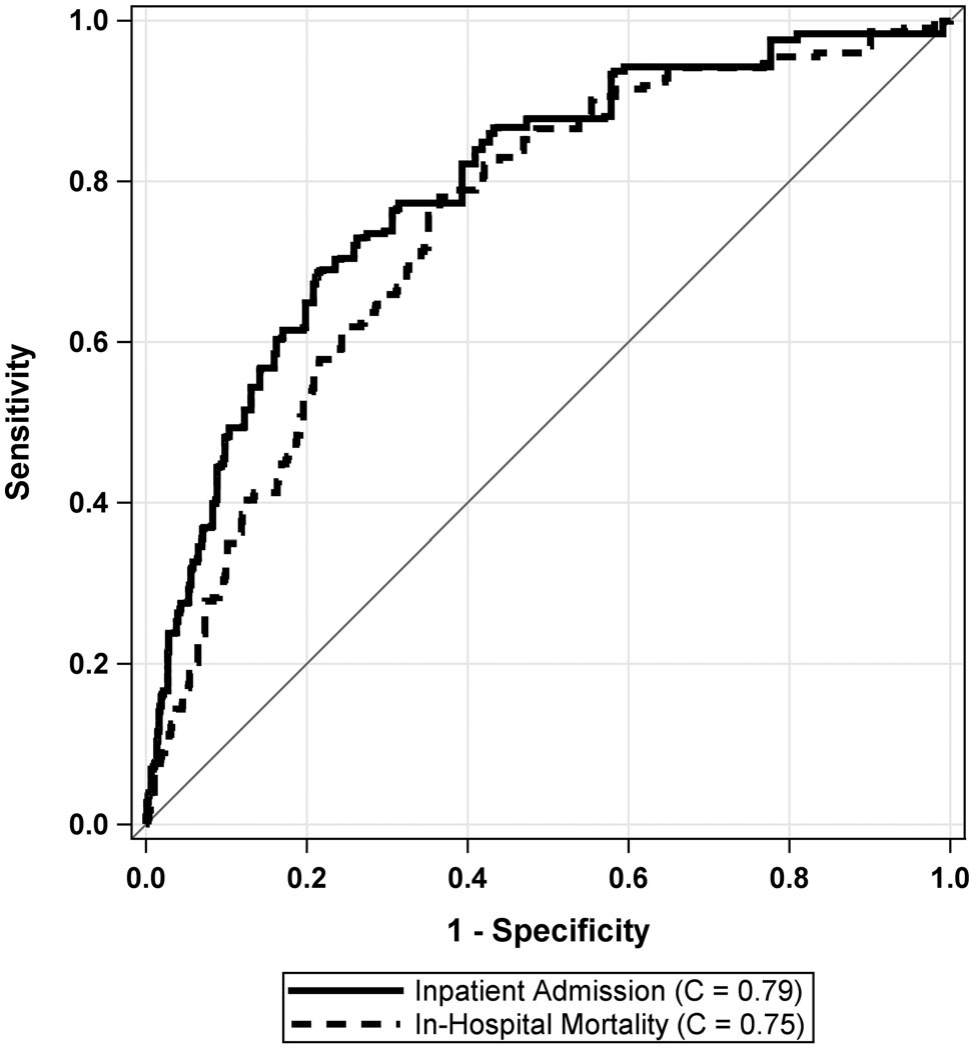
Receiver operating characteristic (ROC) curves for prediction of admission and in-hospital mortality using the COVID-19 Risk of Complications score in a linear fashion.

The cross-validated C-Statistics for the external validation cohort showed good discrimination for both Admission (*C* = 0.79 (95% CI: 0.77, 0.81)) and in-hospital mortality (*C* = 0.75 (95% CI: 0.71, 0.78)). Figure 1 shows the ROC curves for both models.

**Figure 2.**
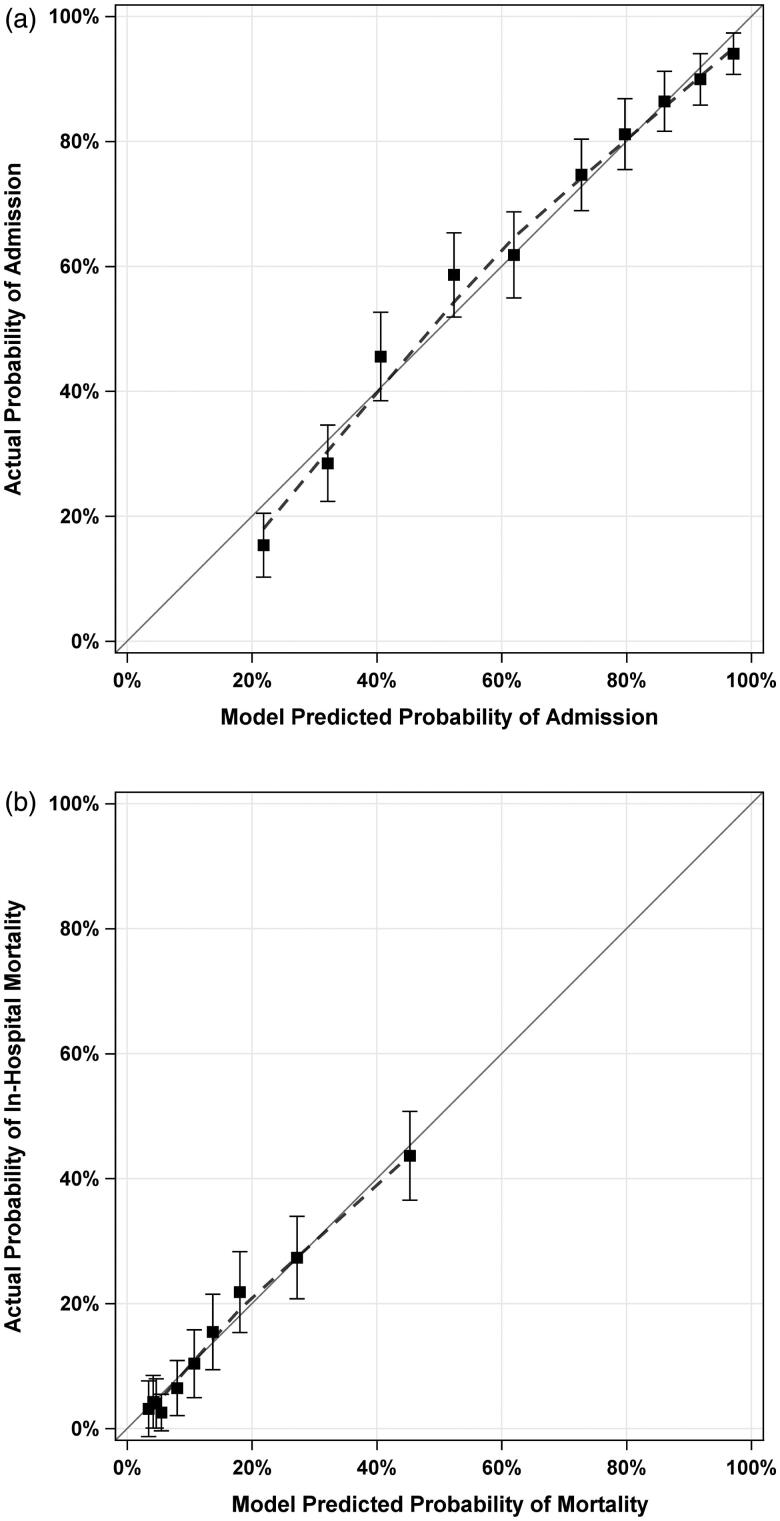
Calibration plots and curves for prediction of admission and in-hospital mortality in external validation cohort. Panel (a) shows the calibration plot for admission in the validation cohort. Panel (b) shows the calibration plot for in-hospital mortality in the Validation Cohort. Dashed lines indicate LOESS-Based Calibration Curves

Calibration for Admission and In-Hospital Mortality are depicted in Figure 2. For admission, the model significantly overestimated the predicted probability of admission for encounters in the lowest decile of predicted probability of admission (< 22%); however, there was no significant overestimation or underestimation for any encounters in any of the other nine deciles. For in-hospital mortality, there was no evidence of significant overestimation or underestimation of the external validation model.

### Other analysis

**Figure 3.**
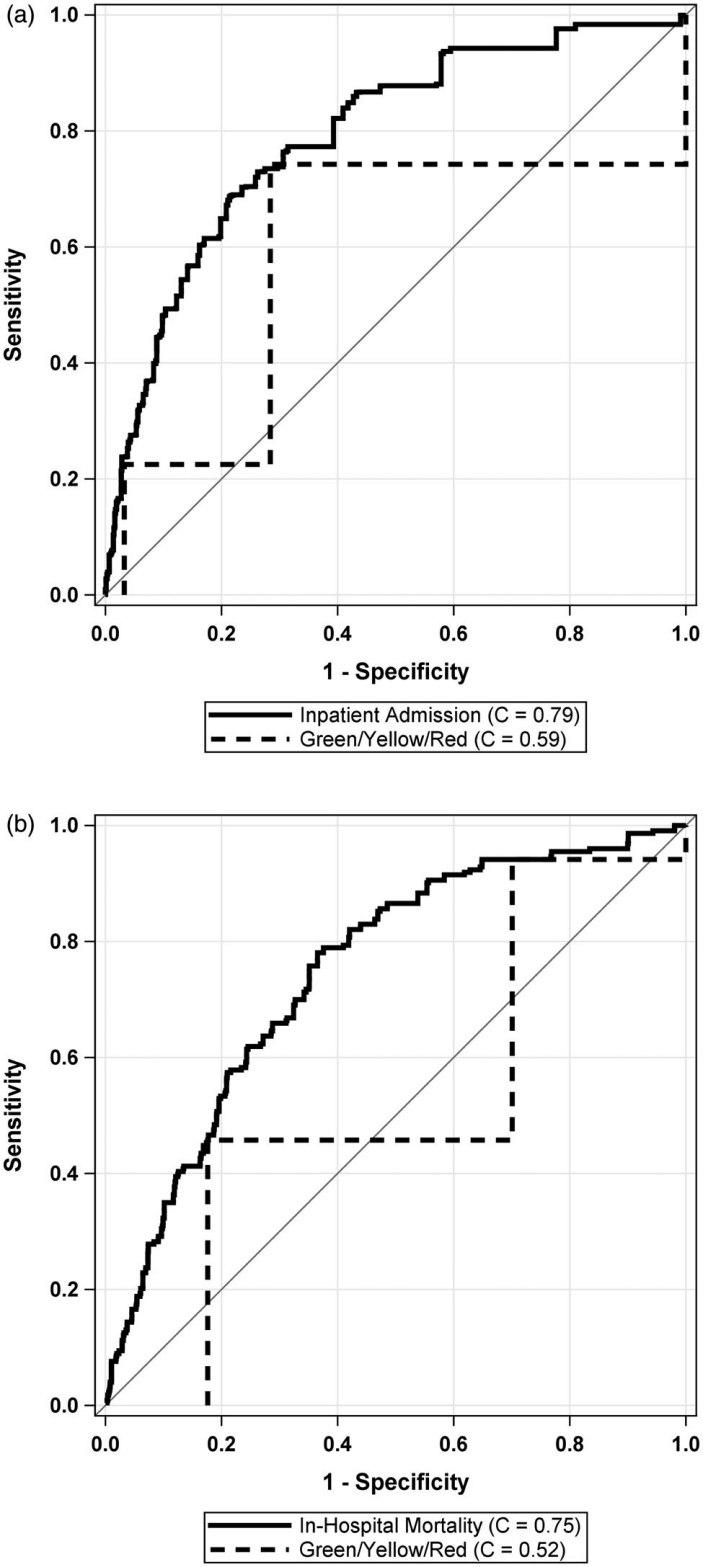
Comparison of external validation model to categorisation of algorithm into Green/Yellow/Red categories. Panel (a) shows the ROC curve for admission in the validation cohort. Panel (b) shows the ROC curve for in-hospital mortality in the validation cohort

Upon examination of the stoplight categories (Green/Yellow/Red), the categorization demonstrates less than good discrimination, in terms of cross-validated C-Statistics, for both Admission (*C* = 0.59 (95% CI: 0.56, 0.61)) and in-hospital mortality (*C* = 0.52 (95% CI: 0.48, 0.56)). Not categorizing the algorithm to Green/Yellow/Red categories led to significantly better discrimination for both admission (C-Statistic increase of 0.20) and in-hospital mortality (C-Statistic increase of 0.23) (both *p* < .0001). Figure 3 compares the ROC between the models that reduce the scoring system to Green/Yellow/Red categories and the full external validation models for admission and in-hospital mortality, respectively.

## Discussion

In this study, we utilized a large multicentric retrospective cohort to validate the COVID-19 Risk of Complications Score for predicting hospital admission and in-hospital mortality of patients diagnosed with COVID-19 when presenting to the emergency department (ED). In general, there was very good calibration of the models predicting admission and in-hospital mortality. For admission, the model significantly overestimated the predicted probability of admission for encounters in the lowest decile of predicted probability of admission (<22%); however, there was no significant overestimation or underestimation for any encounters in any of the other nine deciles. For in-hospital mortality, there was no evidence of significant overestimation or underestimation. The risk score demonstrated satisfactory discriminatory ability for both outcomes as demonstrated by the AUCs of 0.79 and 0.75, respectively. Categorizing patients into stoplight categories (Green/Yellow/Red) proposed by tool developers, in contrast to using the score in a linear fashion, proved unsatisfactory discriminatory value for hospital admission and mortality, demonstrated by AUCs of 0.59 and 0.52 respectively. Contributing factors to the latter observation include a discrepancy between optimal cut-offs for outcomes in our cohort (score of 2 and 4 for hospital admission and mortality, respectively) and the proposed category cut-offs (0–2 green, 3–5 yellow, 6–15 red). Additionally, different risk score constituents had variable predictive abilities for outcomes and hence using uniform weights to score these constituents may affect the overall predictive ability of the model. An example of that effect is evident contrasting risk posed by being male (AOR: 1.76, 95% CI: 1.42,2.19) to having end-stage renal disease (ESRD) (AOR: 3.11,95% CI:1.16,8.31) (Supplementary Appendix tables).

The variables constituting the tool have been reported as risk factors for severe COVID-19 illness or mortality, are constituents of other well validated prognostic tools such as the Charlson Comorbidity Index (CCI), or are mortality predictors for other respiratory illnesses [26–35]. Older age in particular heralds worse outcomes in COVID-19 patients in an incremental [26,30,32,34,35]. On multivariable analysis of our cohort, we found that in addition to older age, end-stage renal disease, chronic pulmonary disease, and nursing home residence were independently predictive of both hospital admission and in-hospital mortality. Additionally, male gender, congestive heart failure, diabetes mellitus, hypertension, and obesity were predictive of admission. However, different risk score constituents had variable predictive abilities for outcomes and hence using uniform weights to score these constituents may affect the overall predictive ability of the model. An example of that effect is evident contrasting risk posed by being male (AOR: 1.76,95% CI: 1.42,2.19) to having end-stage renal disease (ESRD) (AOR: 3.11,95% CI:1.16,8.31) (Supplementary Appendix tables).

The COVID-19 Risk of Complications Score can be easily distributed and readily accessible to a large portion of United States (US) healthcare providers, due to the availability of risk score constituents in the EHR, the automatic computation of the score, and the wide prevalence of Epic EHR in US healthcare systems [21,22]. These factors offer an advantage compared to recently published prediction tools [18] that involve web-based calculators requiring physicians to manually input factors and allow for better generalizability in the United States. Following validation with other external cohorts and after further optimisation of cut-offs, this tool could have significant implications in triaging patients in the outpatient setting for ED referral and in the ED for hospital admission. Patients with higher mortality risk could then be triaged to centres with more available intensive care unit (ICU) beds and advanced oxygenation modalities such as extracorporeal membrane oxygenation (ECMO) in anticipation of worse outcomes. These patients may benefit from earlier or more aggressive medical or procedural interventions such as specific pharmacologic therapy or early prone positioning.

This risk score could be instrumental to identifying higher risk COVID-19 patients that may benefit from close follow-up after discharge. The role of close follow-up in reducing readmission rates in patients with heart failure and cirrhosis is well described [36–38]. Additionally, a longer time to follow-up after hospital discharge is associated with worse outcomes in patients with community acquired pneumonia (CAP) [39]. Tele-visits or provision of monitoring modalities such as ambulatory oximetry might be beneficial to reduce readmissions and improve outcomes. Further analysis should focus on investigating the impact of the risk score usage in decreasing the insurance/healthcare expenditure for COVID-19 patients.

Our study has several limitations. Limitations relating to the tool include the absence of an initial validation cohort for its constituents resulting in uniform scoring weights of different risk factors, and bias created by missing variables reported in other studies as multivariate predictors of outcomes such as imaging findings, levels of C-reactive protein (CRP), lactate dehydrogenase, D-Dimer, and absolute lymphocyte counts [17,18,40]. Limitations relating to our cohort include its retrospective nature, not evaluating mortality in outpatients if it happened outside of our health system, limited outcome data of patients transferred to other facilities, and not analysing time-based outcomes. Additionally, our cohort is limited to the available data in our health system. Statistical limitations included inability to analyse pregnancy, congenital heart disease and end-stage liver disease in the model due to complete separation of these variables. Attempting a penalized regression did not ameliorate these separation effects.

In conclusion, the COVID-19 Risk of Complications Score is a promising, easily distributable, and EHR integrated tool for prediction of hospital admission and in-hospital mortality of COVID-19 patients. However, further refinement of the risk score is required prior to widespread reliance on its use. Future steps should include: (a) validation with other cohorts to assess optimal category cut-offs, (b) evaluation for additional stoplight categories, (c) consideration of different weights of risk score constituents based on their predictive ability, and (d) validation in prospective cohorts with longer follow-up times and with time-based data from symptom onset to outcomes to evaluate time-based outcomes (i.e. time to mortality or hospital admission).

## Supplementary Material

Supplemental Material

## References

[1] Coronaviruses, Including SARS and MERS | Red Book^®^ 2018 | Red Book Online | AAP Point-of-Care-Solutions. [cited 2020 May 18]. Available from: https://redbook.solutions.aap.org/chapter.aspx?sectionid=189640073&bookid=2205.

[2] Park SE. Epidemiology, virology, and clinical features of severe acute respiratory syndrome -coronavirus-2 (SARS-CoV-2; Coronavirus Disease-19)). Clin Exp Pediatr. 2020;63(4):119–124. 32252141 10.3345/cep.2020.00493 PMC7170784

[3] Coronavirus (COVID-19) events as they happen. [cited 2020 May 18]. Available from: https://www.who.int/emergencies/diseases/novel-coronavirus-2019/events-as-they-happen.

[4] COVID-19 Map - Johns Hopkins Coronavirus Resource Center. [cited 2020 Apr 23]. Available from: https://coronavirus.jhu.edu/map.html.

[5] Kampf G, Todt D, Pfaender S, et al. Persistence of coronaviruses on inanimate surfaces and their inactivation with biocidal agents. J Hosp Infect. 2020;104(3):246–251. 32035997 10.1016/j.jhin.2020.01.022 PMC7132493

[6] van Doremalen N, Bushmaker T, Morris DH, et al. Aerosol and Surface Stability of SARS-CoV-2 as Compared with SARS-CoV-1. N Engl J Med. 2020;382(16):1564–1567. 32182409 10.1056/NEJMc2004973 PMC7121658

[7] Morawska L, Cao J. Airborne transmission of SARS-CoV-2: the world should face the reality. Environ Int. 2020;139:105730. 32294574 10.1016/j.envint.2020.105730 PMC7151430

[8] Guan W, Liang W, Zhao Y, et al. Comorbidity and its impact on 1590 patients with Covid-19 in China: A Nationwide Analysis. *Eur Respir J*. Published online March 26, 2020:2000547. doi:10.1183/13993003.00547-2020 PMC7098485 32217650

[9] Richardson S, Hirsch JS, Narasimhan M, and the Northwell COVID-19 Research Consortium, et al. Presenting Characteristics, Comorbidities, and Outcomes Among 5700 Patients Hospitalized With COVID-19 in the New York City Area. JAMA. 2020;323(20):2052. 32320003 10.1001/jama.2020.6775 PMC7177629

[10] Huang C, Wang Y, Li X, et al. Clinical features of patients infected with 2019 novel coronavirus in Wuhan, China. Lancet. 2020;395(10223):497–506. 31986264 10.1016/S0140-6736(20)30183-5 PMC7159299

[11] Wu Z, McGoogan JM. Characteristics of and important lessons from the Coronavirus Disease 2019 (COVID-19) outbreak in China: summary of a report of 72–314 cases from the Chinese Center for Disease Control and Prevention. JAMA. 2020;323(13):1239–1242. 32091533 10.1001/jama.2020.2648

[12] Jean SS, Lee PI, Hsueh PR. Treatment options for COVID-19: The reality and challenges. *J Microbiol Immunol Infect*. 2020;53(3):436-443. 10.1016/j.jmii.2020.03.034 PMC7129535 32307245

[13] Cao B, Wang Y, Wen D, et al. A Trial of Lopinavir-Ritonavir in Adults Hospitalized with Severe Covid-19. N Engl J Med. 2020;382(19):1787–1799. 32187464 10.1056/NEJMoa2001282 PMC7121492

[14] Geleris J, Sun Y, Platt J, et al. Observational study of hydroxychloroquine in hospitalized patients with Covid-19. N Engl J Med. 2020;382(25):2411–2418. 32379955 10.1056/NEJMoa2012410 PMC7224609

[15] NIH Clinical Trial Shows Remdesivir Accelerates Recovery from Advanced COVID-19 | NIH: National Institute of Allergy and Infectious Diseases. [cited 2020 May 19]. Available from: https://www.niaid.nih.gov/news-events/nih-clinical-trial-shows-remdesivir-accelerates-recovery-advanced-covid-19.

[16] Kissler SM, Tedijanto C, Goldstein E, et al. Projecting the transmission dynamics of SARS-CoV-2 through the postpandemic period. Science. 2020;368(6493):eabb5793. 32291278 10.1126/science.abb5793 PMC7164482

[17] Wynants L, Van Calster B, Bonten MMJ, et al. Prediction models for diagnosis and prognosis of covid-19 infection: Systematic review and critical appraisal. BMJ. 2020;369:m1328. 10.1136/bmj.m1328 PMC7222643 32265220

[18] Yan L, Zhang H-T, Goncalves J, et al. An interpretable mortality prediction model for COVID-19 patients. Nat Mach Intell. 2020;2(5):283–286.

[19] About Us | Beaumont Health. [cited 2020 May 14]. Available from: https://www.beaumont.org/about-us.

[20] Clinical management of severe acute respiratory infection when COVID-19 is suspected. [cited 2020 Apr 23]. Available from: https://www.who.int/publications-detail/clinical-management-of-severe-acute-respiratory-infection-when-novel-coronavirus-. (ncov)-infection-is-suspected.

[21] Software | Epic. [cited 2020 May 14]. Available from: https://www.epic.com/software.

[22] All of US News’ top 20 hospitals use Epic. [cited 2020 May 14]. Available from: https://www.beckershospitalreview.com/ehrs/all-of-us-news-top-20-hospitals-use-epic.html.

[23] Information for Healthcare Professionals: COVID-19 and Underlying Conditions | CDC. [cited 2020 May 17]. Available from: https://www.cdc.gov/coronavirus/2019-ncov/hcp/underlying-conditions.html.

[24] Hosmer DW, Jr, Lemeshow S. Applied logistic regression. 2nd ed. New York; Wiley, 2000.

[25] Austin PC, Steyerberg EW. Graphical assessment of internal and external calibration of logistic regression models by using loess smoothers. Stat Med. 2014;33(3):517–535. 24002997 10.1002/sim.5941 PMC4793659

[26] Report of the WHO-China Joint Mission on Coronavirus Disease 2019 (COVID-19). [cited 2020 May 18]. Available from: https://www.who.int/publications-detail/report-of-the-who-china-joint-mission-on-coronavirus-disease-2019-(covid-19. ).

[27] Charlson ME, Pompei P, Ales KL, et al. A new method of classifying prognostic comorbidity in longitudinal studies: development and validation. J Chronic Dis. 1987;40(5):373–383. 3558716 10.1016/0021-9681(87)90171-8

[28] Ewig S, Bauer T, Richter K, et al. Prediction of in-hospital death from community-acquired pneumonia by varying crb-age groups. Eur Respir J. 2013;41(4):917–922. 22903962 10.1183/09031936.00065212

[29] Cher EWL, Allen JC, Howe T, et al. Comorbidity as the dominant predictor of mortality after hip fracture surgeries. Osteoporos Int. 2019;30(12):2477–2483. 31451838 10.1007/s00198-019-05139-8

[30] Shi Y, Yu X, Zhao H, et al. Host susceptibility to severe COVID-19 and establishment of a host risk score: Findings of 487 cases outside Wuhan. Crit Care. 2020;24(1):108. 10.1186/s13054-020-2833-7 PMC7081524 32188484

[31] Yong EL, Ganesan G, Kramer MS, et al. Risk factors and trends associated with mortality among adults with hip fracture in Singapore. JAMA Netw Open. 2020;3(2):e1919706. 32058551 10.1001/jamanetworkopen.2019.19706 PMC12124694

[32] Caramelo F, Ferreira N, Oliveiros B. Estimation of risk factors for COVID-19 mortality - preliminary results. *medRxiv*. 2020;19:2020.02.24.20027268.

[33] Zhou F, Yu T, Du R, et al. Clinical course and risk factors for mortality of adult inpatients with COVID-19 in Wuhan, China: a retrospective cohort study. Lancet. 2020;395(10229):1054–1062. 32171076 10.1016/S0140-6736(20)30566-3 PMC7270627

[34] DeCaprio D, Gartner JA, Burgess T, et al. Building a COVID-19 Vulnerability Index. medRxiv. 2020;2020:03–16.

[35] Xie J, Hungerford D, Chen H, et al. Development and external validation of a prognostic multivariable model on admission for hospitalized patients with COVID-19. *medRxiv*. Published online April 7, 2020:2020.03.28.20045997.

[36] Hernandez AF, Greiner MA, Fonarow GC, et al. Relationship between early physician follow-up and 30-day readmission among medicare beneficiaries hospitalized for heart failure. JAMA - J Am Med Assoc. 2010;303(17):1716–1722. 10.1001/jama.2010.533 20442387

[37] Tong L, Arnold T, Yang J, et al. The association between outpatient follow-up visits and all-cause non-elective 30-day readmissions: a retrospective observational cohort study. PLoS One. 2018;13(7):e0200691. 30016341 10.1371/journal.pone.0200691 PMC6049937

[38] Rao BB, Sobotka A, Lopez R, et al. Outpatient telephonic transitional care after hospital discharge improves survival in cirrhotic patients. World J Hepatol. 2019;11(8):646–655. 31528247 10.4254/wjh.v11.i8.646 PMC6717714

[39] Restrepo MI, Faverio P, Anzueto A. Long-term prognosis in community-acquired pneumonia. Curr Opin Infect Dis. 2013;26(2):151–158. 23426328 10.1097/QCO.0b013e32835ebc6d PMC4066634

[40] Wu C, Chen X, Cai Y, et al. Risk factors associated with acute respiratory distress syndrome and death in patients with coronavirus disease 2019 pneumonia in Wuhan, China. JAMA Intern Med. 2020;180(7):934. 32167524 10.1001/jamainternmed.2020.0994 PMC7070509

