# Supplementary material for: External validation of a clinical risk score to predict hospital admission and in-hospital mortality in COVID-19 patients
Source: Ann Med. 2020 Oct 9;53(1):78–86. doi: 10.1080/07853890.2020.1828616 (PMC7877986; doi:10.1080/07853890.2020.1828616)
Supplement: Supplemental Material [file IANN_A_1828616_SM3699.docx]

**Appendix (Supplementary Material):**

**Appendix Table 1: Summary of the risk assessment tool scoring**

| Risk Factor | Points |
| --- | --- |
| Is immunocompromised | 1 point received if any of the criteria are met:   - HIV diagnosis - Currently receiving chemotherapy - Has an iatrogenic immunosuppression diagnosis - Taking immunosuppressant drugs |
| Age | - <60 years old: 0 points - 60 and 69 years old: 1 point - 70 and 79 years old: 2 points - 80 years or older: 3 points |
| Sex | Male sex: 1 point |
| Congestive Heart Failure | 1 point if present |
| Congenital Heart Disease | 1 point if present |
| End-stage Renal Disease | 1 point if present |
| End-stage Liver Disease | 1 point if present |
| Chronic pulmonary disease | 1 point if present |
| Diabetes | 1 point if present |
| Hypertension | 1 point if present |
| Obesity | 1 point if present |
| Nursing home residence | 1 point if met |
| Pregnancy status | 1 point if met |
| Overall score categories |  |
| Green | 0-2 points |
| Yellow | 3-5 points |
| Red | 6-15 points |

**Appendix Table 2: Univariate and Multivariate Correlates of Admission in External Validation Cohort Data**

|  |  | **Univariate** | | **Multivariate** | |
| --- | --- | --- | --- | --- | --- |
| Age | | **OR (95% CI)** | **P-Value** | **AOR (95% CI)** | **P-Value** |
|  | 60 - 69 years old | 2.29 (1.80, 2.91) | < 0.0001 | 1.69 (1.28, 2.24) | 0.0002 |
|  | 70 - 79 years old | 3.67 (2.70, 4.98) | < 0.0001 | 2.45 (1.71, 3.51) | < 0.0001 |
|  | 80 or older | 9.24 (5.54, 15.4) | < 0.0001 | 6.39 (3.61, 11.3) | < 0.0001 |
|  | < 60 years old | Reference Group |  | Reference Group |  |
| Legal Sex | |  |  |  |  |
|  | Male | 1.39 (1.16, 1.67) | 0.0003 | 1.76 (1.42, 2.19) | < 0.0001 |
|  | Female | Reference Group |  | Reference Group |  |
| Immunocompromised | |  |  |  |  |
|  | Yes | 1.55 (0.87, 2.77) | 0.1399 | 0.75 (0.38, 1.46) | 0.3959 |
|  | No | Reference Group |  | Reference Group |  |
| Congestive Heart Failure | |  |  |  |  |
|  | Yes | 8.12 (4.24, 15.6) | < 0.0001 | 2.17 (1.06, 4.42) | 0.0339 |
|  | No | Reference Group |  | Reference Group |  |
| Congenital Heart Disease | |  |  |  |  |
|  | Yes | 0.28 (0.03, 3.14) | 0.3050 | 0.11 (0.01, 1.24) | 0.0744 |
|  | No | Reference Group |  | Reference Group |  |
| Coronary Artery Disease | |  |  |  |  |
|  | Yes | 4.13 (2.76, 6.17) | < 0.0001 | 0.98 (0.60, 1.58) | 0.9191 |
|  | No | Reference Group |  | Reference Group |  |
| End-Stage Renal Disease | |  |  |  |  |
|  | Yes | 6.63 (2.64, 16.6) | < 0.0001 | 3.11 (1.16, 8.31) | 0.0238 |
|  | No | Reference Group |  | Reference Group |  |
| End-Stage Liver Disease | |  |  |  |  |
|  | Yes | 2.27 (0.25, 20.3) | 0.4623 | 0.72 (0.06, 8.12) | 0.7910 |
|  | No | Reference Group |  | Reference Group |  |
| Chronic Pulmonary Disease | |  |  |  |  |
|  | Yes | 3.94 (3.06, 5.09) | < 0.0001 | 2.55 (1.92, 3.39) | < 0.0001 |
|  | No | Reference Group |  | Reference Group |  |
| Diabetes | |  |  |  |  |
|  | Yes | 4.69 (3.63, 6.05) | < 0.0001 | 2.29 (1.71, 3.07) | < 0.0001 |
|  | No | Reference Group |  | Reference Group |  |
| Hypertension | |  |  |  |  |
|  | Yes | 5.21 (4.26, 6.37) | < 0.0001 | 2.19 (1.71, 2.79) | < 0.0001 |
|  | No | Reference Group |  | Reference Group |  |
| Obesity | |  |  |  |  |
|  | Yes | 2.74 (2.27, 3.30) | < 0.0001 | 2.50 (2.00, 3.13) | < 0.0001 |
|  | No | Reference Group |  | Reference Group |  |
| Nursing Home Residence | |  |  |  |  |
|  | Yes | 8.93 (4.13, 19.3) | < 0.0001 | 3.31 (1.44, 7.59) | 0.0047 |
|  | No | Reference Group |  | Reference Group |  |
| Pregnant | |  |  |  |  |
|  | Yes | 0.17 (0.05, 0.62) | 0.0071 | 0.47 (0.12, 1.92) | 0.2949 |
|  | No | Reference Group |  | Reference Group |  |

**Appendix Table 3: Univariate and Multivariate Correlates of In-Hospital Mortality in External Validation Cohort Data**

|  |  | **Univariate** | | **Multivariate** | |
| --- | --- | --- | --- | --- | --- |
|  |  | **OR (95% CI)** | **P-Value** | **AOR (95% CI)** | **P-Value** |
| Age | |  |  |  |  |
|  | 60 - 69 years old | 2.77 (1.82, 4.21) | < 0.0001 | 2.54 (1.62, 3.97) | < 0.0001 |
|  | 70 - 79 years old | 4.76 (3.14, 7.20) | < 0.0001 | 4.45 (2.79, 7.11) | < 0.0001 |
|  | 80 or older | 5.38 (3.45, 8.40) | < 0.0001 | 5.34 (3.15, 9.06) | < 0.0001 |
|  | < 60 years old | Reference Group |  | Reference Group |  |
| Legal Sex | |  |  |  |  |
|  | Male | 0.96 (0.72, 1.28) | 0.7905 | 1.20 (0.87, 1.65) | 0.2636 |
|  | Female | Reference Group |  | Reference Group |  |
| Immunocompromised | |  |  |  |  |
|  | Yes | 2.40 (1.25, 4.62) | 0.0089 | 1.92 (0.95, 3.91) | 0.0700 |
|  | No | Reference Group |  | Reference Group |  |
| Congestive Heart Failure | |  |  |  |  |
|  | Yes | 2.08 (1.38, 3.15) | 0.0005 | 1.13 (0.69, 1.84) | 0.6348 |
|  | No | Reference Group |  | Reference Group |  |
| Coronary Artery Disease | |  |  |  |  |
|  | Yes | 2.32 (1.63, 3.31) | < 0.0001 | 1.04 (0.68, 1.59) | 0.8654 |
|  | No | Reference Group |  | Reference Group |  |
| End-Stage Renal Disease | |  |  |  |  |
|  | Yes | 2.18 (1.21, 3.29) | 0.0097 | 2.39 (1.22, 4.70) | 0.0116 |
|  | No | Reference Group |  | Reference Group |  |
| Chronic Pulmonary Disease | |  |  |  |  |
|  | Yes | 3.66 (2.71, 4.93) | < 0.0001 | 3.46 (2.50, 4.76) | < 0.0001 |
|  | No | Reference Group |  | Reference Group |  |
| Diabetes | |  |  |  |  |
|  | Yes | 1.66 (1.24, 2.22) | 0.0007 | 1.31 (0.93, 1.83) | 0.1170 |
|  | No | Reference Group |  | Reference Group |  |
| Hypertension | |  |  |  |  |
|  | Yes | 1.49 (1.09, 2.05) | 0.0132 | 0.70 (0.48, 1.02) | 0.0607 |
|  | No | Reference Group |  | Reference Group |  |
| Obesity | |  |  |  |  |
|  | Yes | 0.96 (0.71, 1.30) | 0.8041 | 1.35 (0.94, 1.92) | 0.1007 |
|  | No | Reference Group |  | Reference Group |  |
| Nursing Home Residence | |  |  |  |  |
|  | Yes | 3.07 (1.99, 4.73) | < 0.0001 | 1.87 (1.15, 3.03) | 0.0113 |
|  | No | Reference Group |  | Reference Group |  |

**Appendix Table 4: Model Predicting Admission and Mortality in Green/Yellow/Red Categories**

|  |  | **OR (95% CI)** | **P-Value** |
| --- | --- | --- | --- |
|  | **Admission Prediction** |  |  |
| COVID-19 Risk of Complications Score Categories | |  |  |
|  | Red (Score 6-15) | 19.1 (12.3, 29.7) | < 0.0001 |
|  | Yellow (Score 3-5) | 5.72 (4.62, 7.08) | < 0.0001 |
|  | Green (Score 0-2) | Reference Group |  |
|  | **Mortality Prediction** |  |  |
|  | COVID-19 Risk of Complications Score Categories |  |  |
|  | Red (Score 6-15) | 13.3 (7.28, 24.4) | < 0.0001 |
|  | Yellow (Score 3-5) | 4.73 (2.62, 8.55) | < 0.0001 |
|  | Green (Score 0-2) | Reference Group |  |
